# The World According to Me: Personal Relevance and the Medial Prefrontal Cortex

**DOI:** 10.3389/fnhum.2013.00341

**Published:** 2013-07-02

**Authors:** Anna Abraham

**Affiliations:** ^1^Department of Community Medicine and Behavioural Sciences, Faculty of Medicine, Kuwait University, Safat, Kuwait; ^2^Department of Clinical Psychology, Justus Liebig University Giessen, Giessen, Germany

**Keywords:** self-referential thinking, spontaneous cognition, reality-fiction distinction, default mode network, self relevance

## Abstract

More than a decade of neuroimaging research has established that anterior and posterior cortical midline regions are consistently recruited during self-referential thinking. These regions are engaged under conditions of directed cognition, such as during explicit self-reference tasks, as well as during spontaneous cognition, such as under conditions of rest. One of the many issues that remain to be clarified regarding the relationship between self-referential thinking and cortical midline activity is the functional specificity of these regions with regard to the nature of self-representation and processing. The functional profile associated with the medial prefrontal cortex (mPFC) is the focus of the current article. What is specifically explored is the idea that personal relevance or personal significance is a central factor that impacts how brain activity is modulated within this cortical midline region. The proactive, imaginative, and predictive nature of function in the mPFC is examined by evaluating studies of spontaneously directed cognition, which is triggered by stimulus-associated personal relevance.

“Reality leaves a lot to the imagination.”

(John Lennon)

More than a decade has passed since the publication of the first neuroimaging study of self-referential thinking (Kircher et al., 2000). The original finding of the involvement of cortical midline structures (CMS), such as medial prefrontal and posterior cingulate areas, have been corroborated numerous times since then (Northoff and Bermpohl, 2004; Qin and Northoff, 2011).

The CMS are part of a larger network of areas, commonly referred to as the default mode network (DMN), which are engaged not only during self-referential thought but also during mental state reasoning, autobiographical memory retrieval, episodic future thinking, and moral decision making (Buckner et al., 2008). This has led to several (sometimes overlapping) hypotheses regarding the function of this network as involving either self-projection (Buckner and Carroll, 2007), scene construction (Hassabis and Maguire, 2009), constructive episodic simulation (Schacter et al., 2008), or self-relatedness evaluation (Legrand and Ruby, 2009).

Among the many unanswered questions in this research domain is the functional specificity of each of these regions vis-à-vis the self, given the wide range of social and self-relevant contexts in which this network is activated. The objective in this article is explore the potential role of the ventral (including anterior) aspects of the medial prefrontal cortex (mPFC; dominantly corresponding to Brodmann Areas 10, 32, 11, and 12) in coding for personal relevance or significance, which can be triggered either externally (explicitly or implicitly stimulus-associated) and/or internally (such as during stimulus-independent thought)^1^.

## Self-Relatedness and the mPFC

The idea that the mPFC is responsive as a function of the degree of self-relatedness is not new. In general, the closer the similarity between oneself and another person, the stronger the activation in the mPFC when processing information related to these protagonists, as well as the more ventral the engagement of the mPFC (Mitchell et al., 2006). This pattern of findings has been reported across a range of contexts in which explicit evaluations concerning self and others are made (van der Meer et al., 2010; Murray et al., 2012).

Much of the discourse in the field focuses on parallels and distinctions between neurocognition underlying such kinds of “explicit self-reference” in comparison to “default mode self-reference” (Whitfield-Gabrieli et al., 2011). The latter occurs under conditions of rest or low cognitive demand. However, the ventral mPFC is also selectively engaged in other contexts that are implicit in nature, and in which no explicit self-relatedness judgments are required (Moran et al., 2009; Seitz et al., 2009; Rameson et al., 2010). For instance, the mPFC was responsive to angry body expressions only when a stranger’s body was directly facing oneself, but not when it was turned away (Grèzes et al., 2012). Research on reputation processing has revealed enhanced activity in the mPFC during self-referential thinking in the presence of observers compared to when the participants were unobserved (Izuma et al., 2010). Findings from the ERP literature have also demonstrated that the degree of self-relevance (low, moderate, high, non-self) associated with names of persons or places that were presented to participants, modulated P2 activity, which indexes enhanced attentional recruitment, and P3 activity, which indexes increased cognitive processing (Chen et al., 2011). Moreover, P3 activity, that was elicited when hearing one’s own name, was found to be positively correlated with the degree of brain activity in the mPFC (Perrin et al., 2005).

Such implicit contexts are not equivalent to those of explicit self-reference and default mode self-reference. This is because they involve stimulus-induced self-reference which leads to spontaneous cognition that is not necessarily task-relevant (in that it is not necessary to process the stimuli in a self-referential manner in order to successfully perform the task). As such contexts are broader than those involving “self-relatedness,” the term “self-relevance” is commonly adopted as it more accurately captures the function associated with ventral mPFC activity. Indeed, this fits with dominant ideas in the field regarding the function of this brain region as mediating the “identification and appraisal of *stimulus-induced* self relevance” (author’s italics) (Schmitz and Johnson, 2006, 2007). In contrast, dorsal regions of the mPFC are held to mediate “cognitive control in the generation of explicitly self-referential decisions” (Schmitz and Johnson, 2007).

One question that arises in this context is whether such identification and appraisal processes that trigger spontaneous cognition are also recruited when evaluating contexts that may be personally significant but where the “self” is not directly involved. How does the concept of self-relevance differ from that of personal relevance?

## The Case for Personal Relevance

The word “self” is immediately suggestive of a strong link to one’s subjective or personal identity, such as the knowledge of one’s abilities and skills, personality attributes, preferences, and so on. The object in question in such cases is the “self as I” or the “self as me” (James, 1891). For instance, answering the question “Would you describe yourself as ambitious?” would more actively require you to evaluate this statement in terms of your own self concept than questions such as “Would you describe Barack Obama as ambitious?” or “Would you describe Cinderella as ambitious?”

The concepts of “self-relatedness” or “self-relevance” may apply *prima facie* in contexts related to highly similar or related others (e.g., one’s mother) as such entities are obviously relevant with regard to one’s own self-identity. However, there are several situations in which the applicability or generalizability of such concepts are not as clear-cut. For instance, my favorite coffee mug may be personally relevant to me, but not necessarily self-relevant in the strict sense of being part of my core self-identity. So the concept of “personal relevance” is not entirely synonymous with that of “self-relevance” as it can be applied to a wider range of situations. So what is evidence is there for the modulation of the ventral mPFC as a function of personal relevance?

Investigations of how we make reality-fiction distinctions provide some insight into this question. In the first of these studies (Abraham et al., 2008), participants were presented with sentences in which a real person engaged with either a known real entity (e.g., George Bush) or a fictional entity (e.g., Cinderella) in informative (e.g., heard about) or interactive contexts (e.g., spoke to). Following this, subjects had to determine whether this scenario was possible or not given the constraints of our real world. Processing information about real people led to activations in two brain regions, the anterior mPFC and the posterior cingulate cortex (PCC).

The findings from this explorative study were interpreted in terms of the functional profile associated with these brain regions. Their engagement was postulated to reflect the stimulus-induced spontaneous access and integration of many different kinds of internally generated information (episodic, self-referential, visceral, etc.). Even in the absence of an externally directed behavioral goal that imposes such demands, this information is *automatically* accessed with the introduction of a familiar entity into one’s stream of consciousness. The greater the familiarity, the higher the personal relevance. Activity in these brain regions was therefore held to be spontaneously modulated by the degree of stimulus-associated personal relevance.

The basic premise was that reality (relative to fiction) is processed in terms of subjectively coded representations in the brain. This was attributed to the fact that, among the major differences between familiar famous people and fictional characters, is the amount of information that we can readily draw upon in reference to them and the frequency with which we encounter information regarding them in our daily lives. For instance, we are regularly bombarded with information concerning famous people through the media. Even if we are not likely to encounter these people in reality, they nevertheless occupy a significant space in our social world, unlike fictional characters.

Moreover, although we can arrive at quite a detailed understanding of a fictional world (such as that of Cinderella), we still have, relatively speaking, very limited information about her world in comparison to what we know about our own world. With a famous real entity, such as George Bush, one has access to different types of information about him: the degree of perceived attractiveness, his position in the social hierarchy, the degree of influence his politics has had on one’s own life and that of others, what moral values he stands for, one’s personal feelings toward him (e.g., like/dislike, respect/irreverence), the last time one saw him on television or read about him in the newspaper, etc. So reading about a familiar entity, via stimulus-induction, leads to the spontaneous access, integration, and coordination of many different kinds of information (e.g., semantic, episodic, emotional, self-referential, evaluative, interoceptive), even if this information is unnecessary for the task at hand. In fact, the role of the mPFC has been documented in research on salience processing and valuation, particularly in the presence of personal involvement (Somerville et al., 2010; Roy et al., 2012).

These *ad hoc* speculations were corroborated in a follow-up fMRI study (Abraham and von Cramon, 2009). Familiar individuals within our sociocultural world, such as celebrities or cultural icons, would be expected to be more relevant to us compared to fictional characters because they occupy a real space in our shared social world. But individuals who are part of our intimate circle of family and friends would be even more personally significant as their actions have a direct bearing on our lives. If the mPFC codes for personal relevance, the activation profile seen in this brain region when processing information concerning friends (high relevance), famous people (medium relevance) and fictional characters (low relevance) should vary accordingly. The fMRI results confirmed these expectations as the anterior and ventral mPFC was most strongly engaged during high relevance contexts (e.g., involving one’s mother), moderately engaged in medium relevance contexts (e.g., involving George Bush) and least engaged in low relevance contexts (e.g., involving Cinderella) (Abraham and von Cramon, 2009).

This ties in well with other work that has shown that ventral aspects of the mPFC are engaged when making judgments about others who are similar to us in terms of sociopolitical views (Mitchell et al., 2006), and who are socially relevant to us (Krienen et al., 2010). Merely considering the perspectives of one’s own preferred candidate relative to that of the opponent prior to the 2008 US presidential elections was reflected in a greater mPFC activity (Falk et al., 2012). Moreover, research in the field of cultural neuroscience has revealed that compared to participants of Western origin, the mPFC was more strongly engaged in Chinese participants, not only during self-referential processing, but also during information processing related to one’s mother (Zhu et al., 2007). The rationale offered for this pattern of findings was that China represents an interdependent culture where the conceptual representations of close others are more personally significant than in the case of independent cultures, such as that of Western Europe. Conceptual representations of close others are hence more tightly coupled to conceptual representations of oneself within the semantic networks of people from interdependent cultures relative to those from independent cultures.

While each of the aforementioned investigations tapped some form of explicit self-reference (self-relatedness or self-relevance based), the reality-fiction distinction studies were implicit investigations of personal relevance (Abraham et al., 2008; Abraham and von Cramon, 2009). This is because neither self-referential nor close-other-referential judgments with reference to one’s self concept were necessary for task completion, and the self concept was not passively or indirectly evoked (through one’s own name or through egocentric perspective taking).

As such, the findings revealed that the anterior ventral mPFC is spontaneously modulated by the degree of stimulus-associated personal relevance. Indeed, this fits with the functional profile associated with this region as the constructive processes orchestrated by anterior regions of the ventral mPFC have been highlighted as “… one of combining elemental units of information – from sensory systems, interoceptive cues, long-term memory – into a gestalt representation of how an organism is situated in its environment, which then drives predictions about future events” (Roy et al., 2012).

A powerful demonstration of this principle was reported in a recent article where the influence of personal significance on perception was investigated by having participants tag neutral shapes (e.g., triangle) with labels for themselves, their best friend, or an unfamiliar other (Sui et al., 2013). Self-tagged responses were associated with greater activity in the ventral mPFC and conferred significant behavioral advantages in terms of response speed. This finding of ventral mPFC involvement even in the context of a novel and arbitrary association between neutral stimuli and personal significance builds on previous work where enhanced memory effects (Cunningham et al., 2011) as well as greater mPFC engagement (Kim and Johnson, 2012) were observed for even transitory self-object associations. Together, these findings correspond well to the idea that personal significance is automatically encoded in the brain and modulates information processing accordingly (Roye et al., 2007).

## Conclusion

The central proposal of this article is that the ventral mPFC is responsive as a function of personal relevance. One of the critical factors to note here is that although the engagement of this brain region is “stimulus-induced,” its function cannot be merely explained in terms of explicit self-relevant or self-related task demands. The ventral mPFC is not only involved in explicit contexts, where subjects generate conscious evaluations of oneself or close others, but also in implicit contexts, where, although self-relevant stimuli are presented, no self-referential judgments need to be made (Abraham and von Cramon, 2009; Moran et al., 2009). This illustrates not only the proactive and predictive nature of information processing in the brain (Bar, 2009; Bubic et al., 2010), but also the fact that stimulus-induced spontaneous modulations of the brain can be used to understand such dynamic facets of neurocognitive function.

## Conflict of Interest Statement

The authors declare that the research was conducted in the absence of any commercial or financial relationships that could be construed as a potential conflict of interest.

## Footnotes

^1^The focus of this paper is limited to frontal cortical midline structures as evidence showing that activity in posterior midline regions, such as the precuneus, posterior cingulate, and retrosplenial cortices, are modulated by self-relatedness, self-relevance, personal relevance, and/or personal significance is not as clear-cut.
